# Echocardiographic and Point-of-Care Ultrasonography (POCUS) Guidance in the Management of the ECMO Patient

**DOI:** 10.3390/jcm13092630

**Published:** 2024-04-30

**Authors:** Stephanie Cha, Megan P. Kostibas

**Affiliations:** Department of Anesthesiology & Critical Care Medicine, Johns Hopkins University School of Medicine, 1800 Orleans Street Suite 6216, Baltimore, MD 21287, USA; mkostib1@jhmi.edu

**Keywords:** ECMO, echocardiography, POCUS, vascular access, ARDS, cardiogenic shock, mechanical circulatory support

## Abstract

Veno-arterial (V-A) and Veno-venous (V-V) extracorporeal membrane oxygenation (ECMO) support is increasingly utilized for acute cardiogenic shock and/or respiratory failure. Echocardiography and point-of-care ultrasonography (POCUS) play a critical role in the selection and management of these critically ill patients, however, there are limited guidelines regarding their application. This comprehensive review describes current and potential application of echocardiography and POCUS for pre-ECMO assessment and patient selection, cannulation guidance with emphasis on dual-lumen configurations, diagnosis of ECMO complications and trouble-shooting of cannula malposition, diagnosis of common cardiac or pulmonary pathologies, and assessment of ECMO weaning appropriateness including identification of the aortic mixing point in V-A ECMO.

## 1. Introduction

Extracorporeal membrane oxygenation (ECMO) has become an integral tool in the care of the critically ill patients with cardiac and/or respiratory failure. Over the last 12 years, the numbers of registered centers worldwide have increased from 187 in 2010 to 583 in 2022, while the number of ECMO applications has increased from 3447 to 18,159 in the same timespan [1]. Although ECMO survival continues to improve, its associated mortality is still nearly 50%, therefore this therapy is often reserved for only the most critical patients. Veno-arterial ECMO (V-A ECMO), should be utilized in cardiogenic shock when conventional medical management and intra-aortic balloon pump (IABP) support have failed. Classically, this is defined by systemic systolic pressure less than 90 mm Hg, urine output < 30 mL/h, blood lactate over 2 mmol/L, venous oxygen saturation (SvO_2_) < 60%, and altered consciousness for 6 h, and unresponsive to optimal treatment [2]. Indications for V-A ECMO include acute myocardial infarction, myocarditis, intoxication with cardiotoxic drugs, end-stage dilated or ischemic cardiomyopathy, hypothermia with cardiocirculatory instability, massive pulmonary embolism, post-cardiotomoy cardiogenic shock, bridge to durable ventricular assist device or transplantation, and following a witnessed arrest with immediate, high-quality cardiopulmonary resuscitation (CPR). In contrast, veno-venous ECMO (V-V ECMO) should be considered in severe, acute, reversible respiratory failure that is refractory to maximal medical management. This is defined as severe acute respiratory distress syndrome (ARDS) and refractory hypoxemia (PaO_2_/FiO_2_ < 80 mmHg) and/or severe hypercapnia (pH < 7.25 with PaCO_2_ ≥ 60 mmHg) after conventional management [3]. Specific clinical conditions can include bacterial or viral pneumonia, acute eosinophilic pneumonia, asthma, diffuse alveolar hemorrhage, chest trauma, inhalational injury, bronchopleural fistula, or ventilatory support as bridge to lung transplant. When considering ECMO support, it is important to evaluate for contraindications and the potential for nonrecovery without a viable plan for decannulation.

There are many configurations for V-A and V-V ECMO, and access may be obtained centrally or peripherally (through a percutaneous or open approach). For V-A ECMO, central cannulation is often employed following post-cardiotomy shock or in those with severe peripheral vascular disease precluding peripheral cannulation. Peripheral cannulation is most often performed via the femoral vessels with the drainage (inflow) cannula in the femoral vein and return (outflow) cannula in the femoral artery [4]. Subclavian and axillary artery access are alternative arterial cannulation sites. Additionally, to limit limb ischemia, a distal perfusion catheter should be placed, ideally in the superficial femoral artery, below the level of the femoral bifurcation [5]. For V-V ECMO, flow is usually limited by cannula size. Basic configuration includes a drainage (inflow) cannula that drains deoxygenated blood from the patient and delivers blood to the ECMO circuit, while the return (outflow) cannula returns oxygenated blood to the patient. This approach may involve access at both femoral veins (bifemoral approach) or internal jugular vein-femoral vein approach. Alternatively, a bicaval dual-lumen, single-site cannula may be placed through the internal jugular vein, providing both drainage and return.

Point of care ultrasound (POCUS), transthoracic echocardiography (TTE), and transesophageal (TEE) are pivotal in the pre-cannulation evaluation, cannulation phase, and post-cannulation/monitoring phase in these patients. In the hands of a skilled provider, these tools provide a safe and efficient bedside tool, that except for TEE, is non-invasive.

## 2. Pre-ECMO Assessment

Prior to placing a patient on ECMO, a POCUS exam can be very helpful in identifying anatomy, selection of appropriate ECMO configuration and cannulation sites, and any other concomitant pathologies (Table 1 and Table 2).

For both V-A and V-V ECMO candidates, vasculature should be evaluated with ultrasound for sizing of cannula, patency, location and trajectory, and potential pathology. Major vascular injury (hemorrhage, arteriovenous fistula formation, thrombosis) may occur with a frequency as high as 15% following ECMO cannulation, and this occurs more commonly following percutaneous V-A than V-V cannulation [6]. In one series, limb ischemia secondary to femoral arterial injury occurred at a rate of 16.9% following peripheral V-A cannulation. Fasciotomy was required in 10.3% of these patients and limb amputation in 4.7% [7]. There is abundant data to suggest reduction in vascular complications with ultrasound guidance of vessel cannulation [8,9]. Ultrasound identification of contraindications to cannulation, such as thrombus, noncompressible vein, plaque, stenosis, or prior stent placement, can avoid failed cannulation attempts or vascular injury. In one series, POCUS-guided Seldinger technique demonstrated a success rate of 88% and 86% in patients undergoing ECMO-cardiopulmonary resuscitation (eCPR) with a first pass success rate > 50%. Factors associated with successful cannulation included femoral artery diameter ≥ 4.5 mm, and left ventricular ejection fraction (LVEF) > 20% [10]. For cannula sizing, the following formula is commonly used: cannula caliber (French (Fr)) = 3 × vessel diameter (mm) [11].

In those presenting with acute respiratory failure needing V-V ECMO, it is important to evaluate for lung pathology via POCUS exam. Pathologies like tension pneumothorax or large pleural effusions are diagnosed quickly and facilitate immediate resolution, avoiding unnecessary delay or aborted ECMO cannulation due patient instability. In addition, a focused cardiac examination including assessment of biventricular and valvular function may inform decision making regarding ECMO strategy. For example, acute left ventricular failure could present with acute pulmonary edema and hypoxia. In this scenario, one should consider V-A ECMO rather V-V ECMO. V-A ECMO should be considered with severely decreased left ventricular systolic function, with an LVEF of 20%, with or without wall motion abnormalities [12].

In the setting of acute respiratory failure, it is well known that up to 30% of these patients can show signs of acute right ventricular (RV) dysfunction [12,13]. Hypoxia, hypercarbia, pulmonary edema, and positive pressure ventilation are all variables that increase pulmonary vascular resistance and can unmask RV dysfunction in the setting of a normally highly compliant, low pressure circulation. In these patients, it might be reasonable to consider V-A ECMO cannulation to allow for right heart decompression and support. Echocardiographic features of acute cor pulmonale include RV dilation, flattening of the interventricular septum, left ventricular hypo-diastolic state, enlarged right atrium, tricuspid regurgitation from dilation, low tricuspid annular plane systolic excursion (TAPSE), low tricuspid annular systolic peak velocity (S’), and free wall hypokinesis. Using the simplified Bernoulli equation, the RV systolic pressure can be calculated by adding the central venous pressure (CVP) to the measure peak velocity of the tricuspid regurgitation jet. While most patients have degree of physiologic tricuspid regurgitation, severe tricuspid regurgitation (TR) can be a clear sign of worsening dysfunction and potentially make V-V ECMO less effective in delivering oxygenated blood to the pulmonary circulation. While rare, tricuspid stenosis will also impair flow of oxygenated blood from the return cannula to the pulmonary circulation.

Assessment of shunts, such as a patent foramen ovale (PFO) is also an important consideration. An undiagnosed PFO could become problematic with weaning ECMO, as an increase in right sided pressures could lead to a right to left shunt, and thereby, hypoxia.

**Figure 1:**
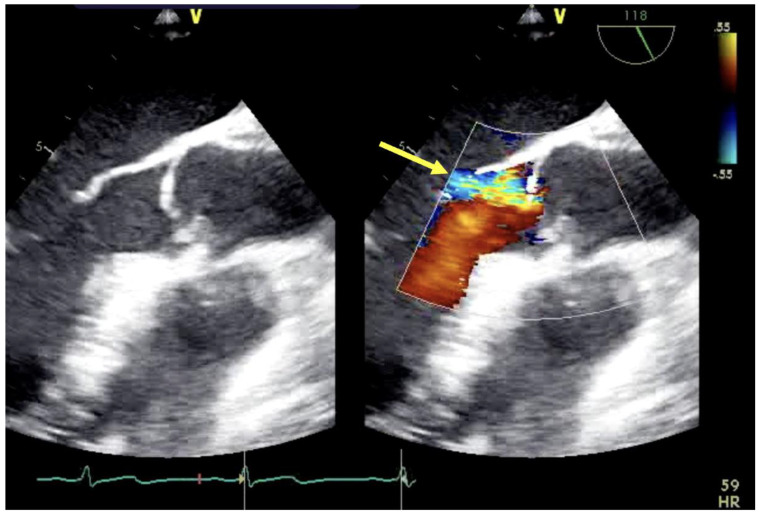
Aortic insufficiency (yellow arrow) demonstrated by TEE mid-esophageal long-axis view.

In those patients being considered for V-A ECMO, POCUS and echocardiographic evaluation is just as critical as for V-V ECMO. Basic evaluations for biventricular function and valve function are important as this can become difficult once decompressed on ECMO. Understanding ventricular size, wall thickness and regional wall abnormalities can be help helpful in guiding management and treatment. Significant aortic insufficiency will result in pulmonary edema and LV distention that leads to increased wall stress, oxygen consumption and therefore ischemia in an already fragile left ventricle. Evaluation for aortic dissection prior to any cannulation is important as its presence is a contraindication to V-A ECMO (Figure 1).

## 3. Procedural/Cannulation Guidance

There are no current recommendations regarding the optimal imaging modality for ECMO cannulation, although the choice to utilize landmarks alone, fluoroscopy, or echocardiography (TTE or TEE) may be guided by urgency of cannulation (i.e., ongoing or imminent cardiac arrest), selected ECMO configuration and involved vasculature, and other patient-related factors that may complicate placement. While use of echocardiography does not consistently prevent peri-cannulation complications, it offers a number of attractive benefits, including pre-cannulation assessment of vasculature to guide cannula size selection and minimization of vascular or cardiac injuries, confirmation of cannula position, avoidance of additional invasive vascular puncture, and avoidance of patient transport to a fluoroscopy suite or operating room, which may minimize time to cannulation as well as medical personnel exposure in infectious contexts (i.e., COVID-19 isolation). The Extracorporeal Life Support Organization (ELSO) guidelines encourage the use of vascular ultrasound to avoid vascular injury or ischemia, and recommend the use of noninvasive TTE prior to the use of TEE to confirm visualization of cannula guidewire and final cannula placement [14]. Support for echocardiography guidance in pediatric literature is even more scarce, although there are few studies to suggest a potential role for TTE to guide jugular cannulation in order to reduce repositioning events and TEE to guide dual-lumen cannulation in patients with pneumomediastinum and massive air leak [15,16].

### 3.1. Echo Considerations

Percutaneous V-V access most often involves cannulation of the internal jugular and/or femoral veins, and ultrasound should visualize the drainage cannula guidewire and final cannula placement in the hepatic inferior vena cava (IVC) and/or superior vena cava (SVC), and the return cannula guidewire and final placement in the right atrium (Table 1). Percutaneous V-A access most often involves cannulation of the femoral artery and vein, and should include visualization of the return cannula in the descending aorta, below the level of the left subclavian artery takeoff. This may be accomplished with TTE and POCUS (suprasternal notch and abdominal aortic views), but it is best visualized with TEE (aortic views). If the axillary artery is utilized as the return arterial vessel, echocardiography should visualize the return cannula guidewire within the descending aorta, and confirm that it does not course through the aortic valve and left ventricular outflow tract (Table 2). Overall and first-pass success rate for vascular cannulation may be improved with vascular POCUS guidance (vs. fluoroscopy or landmark technique), even in vulnerable populations such as those presenting in cardiogenic shock or eCPR [17]. Similarly, cannulation time was demonstrated to be faster under vascular POCUS guidance vs. landmark technique in patients requiring urgent cannulation. In a series of patients with in-hospital cardiac arrest presenting for catheterization for acute myocardial ischemia, cannulation under ultrasound guidance was achieved in 82 min versus 128 min under the landmark technique [18].

### 3.2. Dual-Lumen Considerations

An alternative cannulation strategy for V-V ECMO involves placement of a dual-lumen cannula (Avalon Elite Catheter by Getinge, Crescent Jugular Dual Lumen Catheter by Medtronic) in the right internal jugular vein. Drainage occurs at SVC and hepatic IVC sites, while blood is returned directly above the tricuspid valve. This configuration may offer advantages including single-site placement, minimization of infectious and vascular complications, improved efficiency of oxygenation via bicaval drainage, improved patient mobility, and the ability to transition from V-A to V-V techniques without additional cannulation or circuit disruption [19].

**Figure 2:**
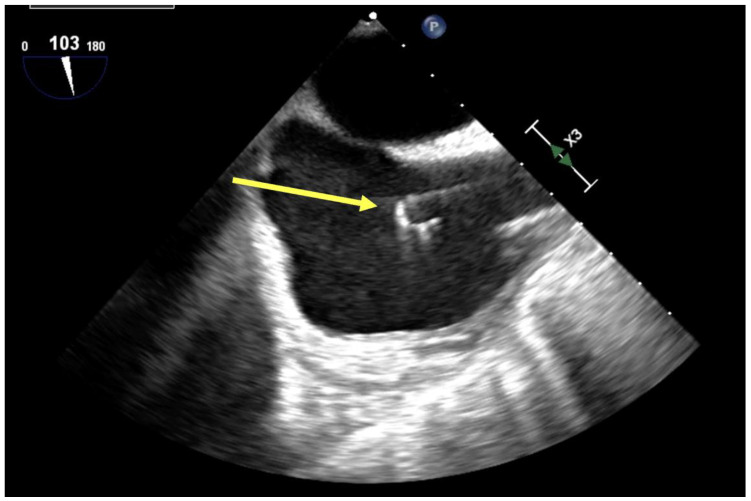
Visualization of J-tip of guidewire (yellow arrow) within right atrium by TEE mid-esophageal bicaval view.

**Figure 3:**
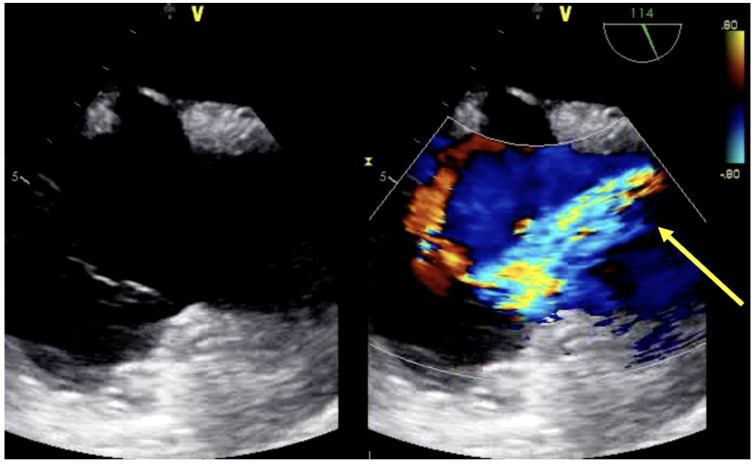
Visualization of oxygenated, return blood flow (yellow arrow) directed above the tricuspid valve by TEE modified bicaval view.

Dual lumen cannula placement occurs under either fluoroscopy or echocardiographic guidance. Cannula markers indicative of outflow sites are confirmed by fluoroscopy. TEE, however, has been well described to guide dual-lumen cannula placement, especially in special populations (COVID-19) to avoid patient transport to a fluoroscopy suite and minimize exposure to medical personnel [20]. TEE guidance is achieved via mid-esophageal bicaval or modified bicaval view. The J-tip of the guidewire must be visualized passing and terminating in the hepatic IVC, avoiding hepatic vein branches or inadvertent passage into the right ventricle (Figure 2). Placement can be further confirmed by demonstration of turbulent flow at cannula drainage pores in the SVC and IVC, and by the turbulent outflow “jet” directed at the tricuspid valve, with minimal divergence or swirling within the right atrium (Figure 3) to avoid complications such as intracardiac injury/right ventricular perforation, hepatic congestion, recirculation, or structural damage [20]. Contrast echocardiography may additionally confirm optimal flow orientation, especially by providing high resolution imagery in patients with significant lung pathology, but its application is less frequently described [21].

TEE guidance for dual-lumen cannulation has been reported with a high success rate of >90% [22,23]. In one series of patients with acute respiratory distress syndrome (ARDS), the success rate was 99% and the repositioning rate was 13% [24]. Additionally, the number of attempts at cannulation has been demonstrated to be equivalent among groups cannulated under TEE vs. fluoroscopy, but TEE offered fewer low-flow and repositioning events [22]. In a series of COVID-19, ARDS patients, TEE again demonstrated high success rate, but up to 38% patients required repositioning. Factors associated with difficult cannulation included extreme body size, wire bending in the right atrium, and prominent eustachian valve [23].

### 3.3. Peri-Cannulation Support

Acute hemodynamic lability, hypoxemia, or even cardiac arrest may be incited by cannulation itself. Often, this is due to profound hypoxemia and/or right ventricular dysfunction in the case of acute respiratory pathology (V-V cannulations) and worsening of acute cardiogenic shock during V-A cannulation. Here, echocardiography may serve as a powerful tool to rapidly identify and manage reversible etiologies for decompensation. Visualization of acute myocardial dysfunction, tamponade, hemothorax, or tension pneumothorax by POCUS have been well described with at least equivalent sensitivity to conventional imaging, and often results in faster time to definitive management [25,26].

## 4. Monitoring of ECMO

### 4.1. V-A

Monitoring of V-A ECMO should include serial assessment of biventricular function. Echocardiography may more accurately estimate cardiac output than alternative monitoring strategies, i.e., overestimation by pulmonary artery catheterization/thermodilution technique due to negative right atrial pressures, and underestimation by pulse contour analysis due to low systemic pulsatility [27]. In addition, echocardiography may identify complications related to ECMO management itself, such as the development of cardiac tamponade due to anticoagulation or cardiac injury.

Most importantly, echocardiography may aid in the assessment of LV unloading to minimize LV distension and myocardial demand, subendocardial ischemia and pulmonary edema, and guide optimal ECMO flow rates. Echocardiographic visualization of left atrial and left ventricular decompression, aortic valve opening, and absence of severe mitral regurgitation or intracardiac spontaneous echo contrast or thrombus may help to confirm appropriate left ventricular decompression [28]. Contrast echocardiography may additionally be helpful in changing ECMO management by detecting LV thrombi, intracardiac masses and identifying LV dysfunction with greater resolution than conventional TEE [21]. However, microspheres associated with contrast administration may trigger or alarm circuit dysfunction, cause shutdown, or even be prone to destruction due to circuit shear forces [21,29].

**Figure 4:**
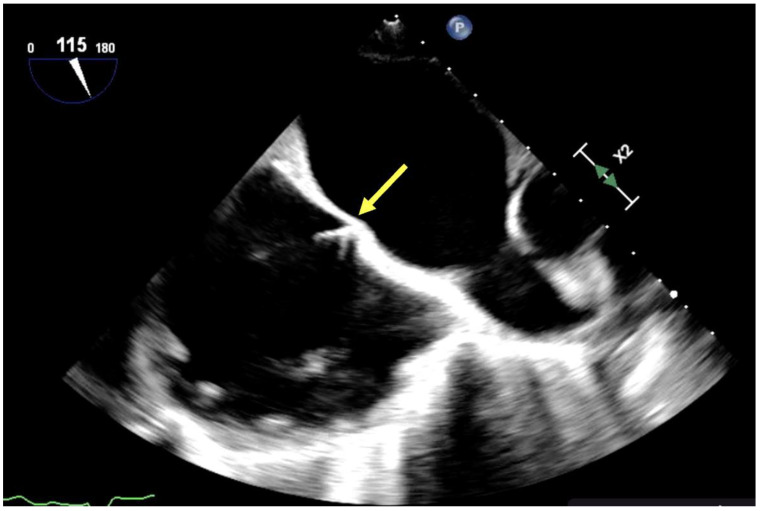
Interatrial “tenting” (yellow arrow) during trans-septal puncture demonstrated by bicaval view on TEE.

**Figure 5:**
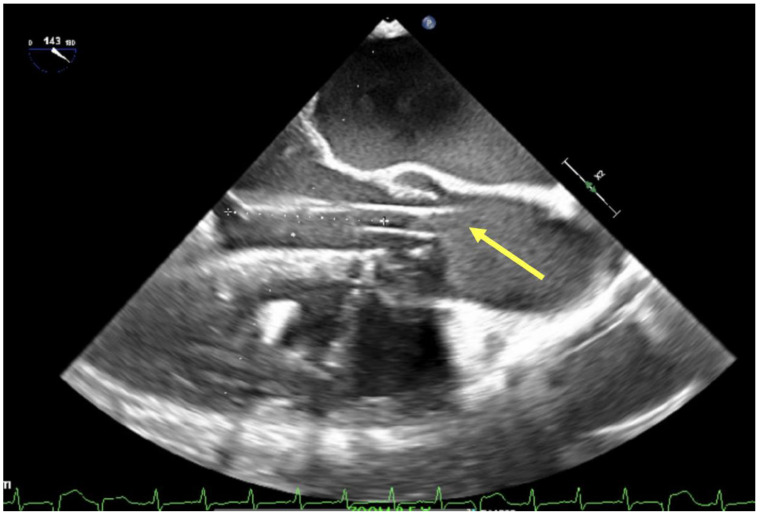
Impella (yellow arrow) visualization by TEE in a midesophageal long-axis aortic valve view.Impella is traversing the aortic valve with distal tip in left ventricle.

If left ventricular unloading is insufficient, placement of an LV vent is indicated and associated with increased probability of ECMO weaning and reduced mortality [28]. Echocardiography may be a useful guide in placement and management of LV vents, regardless of LV venting strategy. LV vents may be placed directly in the LV apex or left atrium (via pulmonary vein) under TEE guidance. In addition, LV venting may be achieved by inter-atrial septostomy. Septostomy is often guided by TEE, which facilitates visualization of interatrial “tenting” and may benefit from en-face 3D image guidance to aid interventionalists with fine intracardiac movements to avoid cardiac or vascular injury (Figure 4) [30]. Intra-aortic balloon pulsation (IABP) is another often utilized strategy to address LV distension via reduction in systemic afterload, and when utilized with ECMO, may improve femoral arterial blood flow rates and overall survival compared with ECMO therapy alone. TEE guidance has been described to guide appropriate placement. IABP guidewire should be visualized 1–2 cm below the left subclavian artery takeoff, and views should confirm absence of new cardiac injury, aortic dissection, or interference with atheromatous disease [31,32,33]. Finally, the LV Impella (Abiomed) is a miniaturized left ventricular assist device which provides continuous drainage of left ventricular blood flow to the ascending aorta. It is often utilized in conjunction with ECMO therapy to provide both LV unloading as well as a transitional form of mechanical circulatory support following ECMO decannulation, and it has been associated with improved survival when utilized in conjunction with ECMO vs. ECMO support alone [34]. TEE is frequently utilized to guide Impella placement, and should demonstrate the Impella tip 3.5–5.5 cm (depending on Impella version) from the aortic annulus on the ventricular side of the aortic valve, as well as confirm absence of consequent aortic dissection, cardiac tamponade, or new aortic valve or mitral valve dysfunction, or patent foramen ovale [35] (Figure 5).

**Figure 6:**
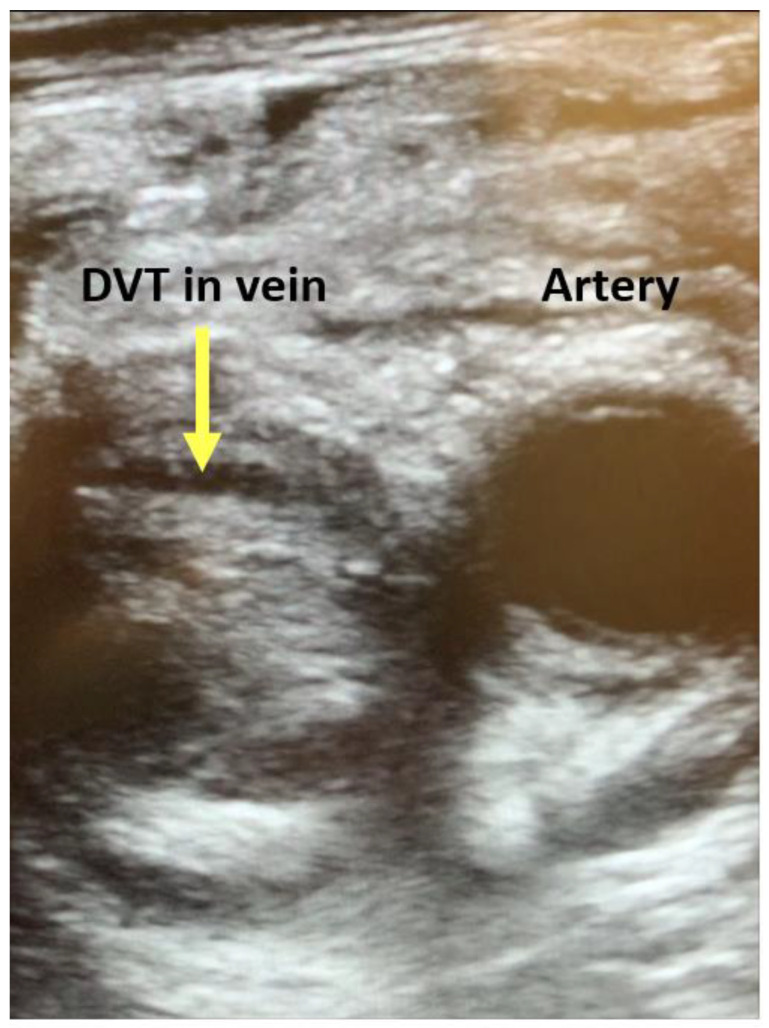
Acute deep venous thrombosis visualized in femoral vein by vascular POCUS.

For cases of isolated RV failure, echocardiography continues to serve as a mainstay for both initial diagnosis and assessment of ongoing management. Acute RV strain characterized by increased RV to LV ratio, abnormal septal motion, tricuspid regurgitation, visualization of right heart thrombus, RV hypokinesis, pulmonary hypertension, elevated RV systolic pressure, reduced TAPSE, McConnell’s sign (RV mid-free wall akinesis with normal apical motion), and “60/60” sign (tricuspid regurgitation jet< 60 mm HG and pulmonary flow acceleration time < 60 ms) has been shown to perform as an adequate “Rule-in” test for pulmonary embolism (PE), with high specificity and low sensitivity [36]. Similarly, visualization of new deep venous thrombosis (DVT) by “2-point” vascular POCUS exam (femoral and popliteal vessel interrogation) in the setting of suspected PE may justify treatment for PE without further testing [37] (Figure 6). In addition, echocardiography and/or POCUS may be useful to guide management decisions, for instance, main pulmonary artery size has been associated with need for surgical embolectomy vs. ECMO and anticoagulation alone [38]. Furthermore, echocardiography and POCUS may direct alternative strategies including catheter-mediated suction embolectomy [39]. Resolution of acute RV dysfunction and main pulmonary artery thrombus vs. transition to chronic RV dysfunction with vascular remodeling may additionally be monitored by echocardiography [36].

### 4.2. V-V

Refractory hypoxemia may occur despite VV ECMO support and may be caused by a number of pathologies, for which echocardiography may serve as a useful diagnostic tool. Cannula malposition may occur with patient movement or changing intrathoracic volume (i.e., development of tension pneumothorax or worsening atelectasis), resulting in inefficient return of oxygenated blood flow. TEE or TTE revisualization of return outflow directed at the tricuspid valve may be required. In addition, malposition may be due to recirculation, which occurs when inflow and outflow cannulas are in close approximation, resulting in a low resistance pathway that bypasses the ECMO circuit. In patients with dual-lumen cannula, recirculation can result from the cannula being either too high in the superior vena cava and right atrium or too deep with all ports within the hepatic IVC. Clinically, this occurs in conjunction with high ECMO flow rates and high venous oxygen saturation, which are often monitored by the ECMO circuit. Echocardiography may also reveal close proximity of drainage and return cannula tips, for instance, visualization of SVC and IVC cannula in close approximation within the right atrium.

Hypovolemia or cannula malposition deep within the hepatic IVC or terminating within a small hepatic vein may cause a low-flow state and hypoxemia, and is suggested by echocardiography confirmation of empty right ventricular chambers and visualization of “suckdown” or collapse around the cannula with consequent obstructed drainage around the drainage cannula tip or termination of the drainage cannula in a distal hepatic venous branch. Similarly, visualization of intra-cannula thrombus may be confirmed as an alternative etiologies of impaired drainage flows.

High cardiac output states seen in developing sepsis, fever, or high body mass index may also cause hypoxemia due to increasing shunt fraction. Echocardiography may be useful in quantifying shunt fraction by estimation of pulmonary blood flow (PBF). This requires measurement of the right ventricular outflow tract (RVOT) diameter (mid-esophageal RV inflow-outflow view), and RVOT velocity time integral (VTI) such that PBF = [piX(RVOT diameter/2)^]XRVOT VTI.

**Figure 7:**
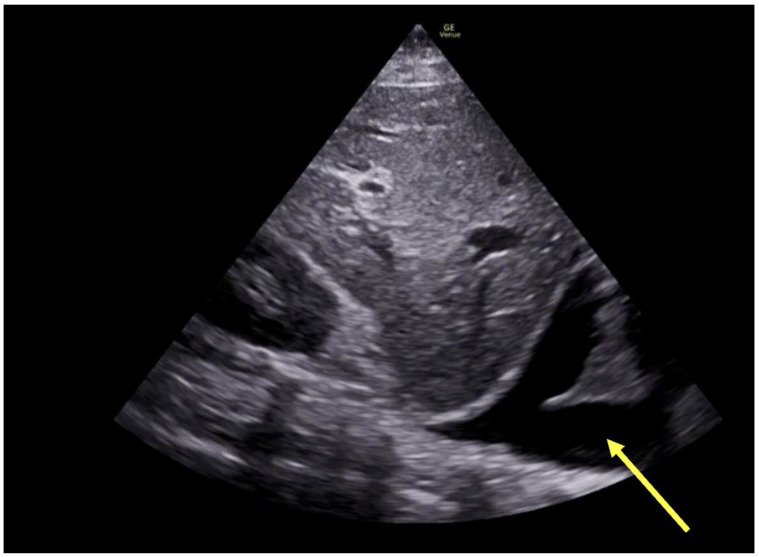
Large pleural effusion (yellow arrow) visualized by POCUS lung examination.

Acute or chronic worsening of lung processes may also cause worsening hypoxemia and/or acute RV dysfunction with consequent remodeling of the pulmonary vasculature [40]. POCUS has been well described as a sensitive exam for detecting new pneumothorax (visualization of “lung point”), hemothorax or large pleural effusion (Figure 7), or consolidation [26]. In addition, cardiac POCUS and/or TEE has been utilized in ARDS patients to detect and grade severity of new acute RV dysfunction, evidenced by RV dilation, estimated pulmonary artery pressure, septal dyskinesis, and RV hypertrophy, which may have implications regarding pharmacologic and/or ventilatory strategy, in addition to prognosis [40].

## 5. ECMO Weaning

### 5.1. V-A

Echocardiography has been well described as a useful adjunct in weaning V-A ECMO to complete liberation or transitioning to alternative mechanical circulatory support [41]. While there are no standardized echocardiography protocols, assessment should demonstrate recovery of cardiac function with reduction in ECMO flow to minimal ranges (1–1.5 L/min). A number of markers have been associated with cardiac recovery and successful V-A ECMO weaning, including LVEF > 20–25%, mitral valve lateral annulus tissue(s′) doppler > 6 cm/s, LVOT VTI > 10 cm/s on 1.5 L of ECMO flow, strain or strain rate > 20% baseline, improvement of lateral e′ velocity, and improvement of tricuspid annular S′ velocity [42]. In addition, with recovery of LV performance in peripheral V-A ECMO, aortic mixing point (where LV stroke volume interfaces with femoral arterial cannula flow) should move distally with identification in the descending aorta. Localization of mixing point has been described utilizing aortic ultrasound and doppler flow measurement [43]. For etiologies of cardiogenic shock and isolated right ventricular failure requiring V-A ECMO, right ventricular function may be similarly assessed via TTE or TEE.

### 5.2. V-V

There is little data to support the use of echocardiography in weaning of V-V ECMO, however, echocardiography may be useful to assess markers of improving right ventricular function and lung aeration. Markers of right ventricular function that may be useful include measurement of right ventricular size, right ventricular fractional area change, and right ventricular strain. In contrast, TAPSE and severity of tricuspid regurgitation have not been well correlated with successful weaning [28].

**Figure 8:**
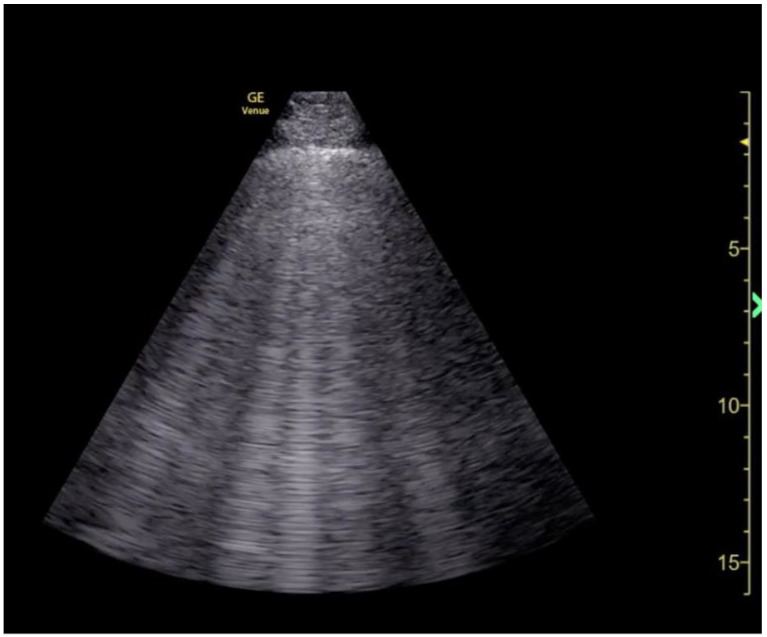
“B-lines” visualized by POCUS lung examination.

Assessment of lung function, however, has been well studied in special populations, including those with decompensated heart failure, end stage renal disease, or requiring intensive care unit admission [44,45]. The presence of pulmonary edema has been well correlated with lung scoring systems through identification of “B-lines”, an ultrasound artifact created by the accumulation of water within the interlobular septa of alveolar spaces, resulting in vertical “B-line” beams originating from the pleura, and extending throughout the depth of visualized lung parenchyma [26] (Figure 8). Most commonly, lung aeration is scored numerically within a regional lung “zone” described according to presence of B-lines or demonstration of consolidation, such that higher scores are assigned to poorer lung aeration. Individual lung zone scores are then summed for a composite lung score. There are several studies which have correlated lung aeration score with conventional imaging (chest X-ray, computed tomography) to assess improving lung volume status and lung compliance in ECMO patients [46,47]. Regional scores of lung zones are then summed for a composite lung score. Similarly, lung ultrasound score has correlated with V-V ECMO weaning success in COVID-19 patients and neonates and survivorship following ECMO-supported ARDS [48,49,50].

## 6. Conclusions and Future Directions

Despite a lack of comprehensive guidelines on the use of echocardiography and POCUS in pre-ECMO assessment, peri-cannulation, management and weaning of ECMO patients, it is clear that the application of these tools is instrumental in caring for these critically ill patients. Daily evaluation with echocardiography, lung ultrasound and vascular ultrasound should be a priority in ECMO patients, assuming the equipment and expertise are available. Ultrasound allows one to gather information with a high diagnostic capacity in an efficient and non-invasive fashion. It has become standard of care in the critical care populations and should be no different in the ECMO population. Consensus guidelines for the use of these modalities is needed for patients being considered for and on ECMO support to improve clinical management, care and outcomes.

## Figures and Tables

**Table 1:** Echocardiographic/POCUS consideration for V-A ECMO.

Pre-Cannulation	Cannulation	Post-Cannulation (Monitoring)
Assessment of biventricular function	Guidance of guidewire and cannula(s) placement	Assessment of biventricular function
Assessment for severe aortic insufficiency	Guidance of LV vent placement	Assessment of LV unloading
Assessment of peripheral vasculature for size, disease, thrombus	Assessment for vascular or intracardiac injury (dissection, tamponade)	Assessment of aortic mixing point
Evaluation for pericardial fluid		Assessment for resolution of cardiogenic shock (i.e., RV failure, resolution of pulmonary artery thrombus)
Assessment for aortic dissection, thrombus		

**Table 2:** Echocardiographic/POCUS considerations for V-V ECMO.

Pre-Cannulation	Cannulation	Post-Cannulation (Monitoring)
Assessment of biventricular function	Guidance of guidewire and cannula(s) placement	Assessment of RV function
Assessment of tricuspid valve disease	Assessment for vascular or intracardiac injury (RV puncture, tamponade), tension pneumothorax, hemothorax	Assessment of lung aeration and/or new lung pathologies
Assessment of peripheral vasculature for size, disease, thrombus	Assessment of return (outflow) “jet”	Confirmation of cannula position
Evaluation for pericardial fluid		Guidance for cannula repositioning
Assessment for aortic dissection, thrombus		
